# Persistence and Active Replication Status of Oropouche Virus in Different Body Sites: Longitudinal Analysis of a Traveler Infected with a Strain Spreading in Latin America

**DOI:** 10.3390/v17060852

**Published:** 2025-06-16

**Authors:** Andrea Matucci, Elena Pomari, Antonio Mori, Silvia Accordini, Natasha Gianesini, Rebeca Passarelli Mantovani, Federico Giovanni Gobbi, Concetta Castilletti, Maria Rosaria Capobianchi

**Affiliations:** 1 Department of Infectious, Tropical Diseases and Microbiology, IRCCS Sacro Cuore Don Calabria Hospital, 37024 Negrar di Valpolicella, Verona, Italy; andrea.matucci@sacrocuore.it (A.M.); elena.pomari@sacrocuore.it (E.P.); antonio.mori@sacrocuore.it (A.M.); silvia.accordini@sacrocuore.it (S.A.); natasha.gianesini@sacrocuore.it (N.G.); rebeca.passarellimantovani@sacrocuore.it (R.P.M.); federico.gobbi@sacrocuore.it (F.G.G.); mrcapobianchi@gmail.com (M.R.C.); 2 Department of Clinical and Experimental Sciences, University of Brescia, 25121 Brescia, Italy; 3 Medicine and Surgery Faculty, Saint Camillus International University of Health Sciences, 00131 Rome, Italy

**Keywords:** Oropouche virus, viral RNA, virus replication, persistent infection, genomic RNA, antigenomic RNA, surrogate of replication

## Abstract

An unprecedented outbreak of Oropouche virus (OROV) is occurring in the Americas, characterized by thousands of confirmed cases and a wide geographical spread, including areas outside the Amazon Basin. Little is known about this neglected arbovirus regarding its pathophysiological aspects and potentially different transmission modes. This study describes the clinical course of a man who returned from a trip to Cuba and presented to our hospital 4 days after the onset of febrile symptoms. The patient was diagnosed with Oropouche fever and was followed for 177 days after the onset of symptoms. We performed a longitudinal investigation of the samples collected from several body sites (whole blood, serum, urine, and semen) with the aim of providing further insights into OROV infection dynamics, using the detection of antigenomic RNA as a marker of active viral replication. Clinical samples that were longitudinally collected over the course of OROV infection showed consistently higher amounts of antigenomic RNA compared to genomic RNA, even after viral clearance from serum. Moreover, our case study showed the persistence of OROV RNA in serum of less than 15 days from the onset of symptoms, as compared to up to one month in urine, three months in semen, and four months in whole blood. Our study suggests that Oropouche virus may persist in an actively replicating state in different body sites for long periods of time, with important implications for transmission dynamics. Furthermore, our results provide a diagnostic indication, suggesting that serum is inferior to both urine and whole blood as preferred diagnostic samples. Further studies are needed to determine the pathogenetic implications of these findings, as they have been derived from a single case and must be confirmed using a larger number of cases.

## 1. Introduction

Oropouche virus (OROV) is an arthropod-borne virus endemic to the Americas, belonging to the genus *Orthobunyavirus* in the *Peribunyaviridae* family [1]. It was first identified in 1955 in a febrile forestry worker in Trinidad and Tobago. OROV is mainly transmitted by the bite of midges (*Culicoides genus*) and causes non-specific symptoms that are usually mild, including fever, chills, headache, and, in some cases, nausea and rash [2]. Since 1955, increasing numbers of OROV infections have been reported in the Amazon region. An unprecedented OROV outbreak emerged in late 2023, characterized by thousands of confirmed cases and a wide geographical spread, including in areas outside of the Amazon Basin [3,4]. In May 2024, the Ministry of Public Health of Cuba reported the first-ever outbreak of OROV disease [5]. On 3 August 2024, the Pan American Health Organization/World Health Organization (PAHO/WHO) raised the epidemiological alert from medium to high [6], following the initial alert issued in February. The first recorded cases diagnosed outside the endemic areas also occurred in 2024, with 19 cases reported in Europe and additional cases identified in the United States [7,8].

The first two cases outside the Americas were diagnosed in Italy at our hospital, the IRCCS Sacro Cuore Don Calabria (IRCCS-SCDC) in Negrar, Verona [9].

OROV disease was identified many years ago, but the recent rise in cases and the spread of the virus to new areas highlight how little is known about its pathophysiological aspects, clinical impact, various modes of transmission, and the different vector capabilities for OROV transmission. Recent studies [10,11] have demonstrated the potential of this virus to induce severe disease and death, with vertical transmission causing miscarriages, fetal deaths, and microcephaly.

In addition, the persistence of OROV infection in different clinical samples has recently been reported, evidenced by the detection of OROV RNA in whole blood for at least three months—albeit at low viral RNA loads [12,13,14]—and in semen up to two months after symptom onset, raising questions regarding potential sexual transmission and emphasizing the need for further research [15,16].

Infectious OROV was isolated from semen 15 days after symptom onset (DSO); however, the active viral replication state in different human body sites has not yet been fully elucidated. In fact, it remains unclear whether the viral RNA—detectable for long periods of time—represents an actively replicating virus or vestigial viral components that are remnants of past replication or passive transport.

Like all members of the *Orthobunyavirus* genus, the OROV genome consists of three negative-sense single-stranded RNA segments (ssRNAs), designated Large (L), Medium (M), and Small (S), according to their respective size. The L segment encodes the RNA polymerase (L protein), M encodes the envelope glycoproteins (Gn and Gc) and non-structural M protein (NSm), and S encodes the nucleocapsid protein (N protein) and, in most orthobunyaviruses, the non-structural S protein (NSs) [1]. As with other peribunyaviruses, the genomic vRNAs are neither polyadenylated nor modified at the 5′ end. Viral mRNAs are also not polyadenylated and are truncated relative to the vRNA. They possess a 5′-methylated cap derived from the host mRNA via “cap snatching”, which is mediated by the endonuclease function of the L protein [1].

The replication cycle of the *Orthobunyavirus* genus is complex and requires the synthesis of an intermediate, complementary, positive-sense ssRNA+ (antigenomic RNA—ag-RNA), which is identical in size to the genomic RNA (g-RNA) [1]. Briefly, after the virus enters the host cell, the genomic RNA (g-RNA) is released into the cytosol, where the RNA-dependent RNA polymerase (RdRp) carried by the infectious viral particles produces the ag-RNA intermediate. This ag-RNA functions both as a template for synthesizing novel g-RNA and as mRNA, directing the synthesis of new viral proteins. Consequently, the novel g-RNA molecules are packaged into progeny viral particles and then released [17,18,19]. Therefore, the presence of ag-RNA molecules is a univocal biomarker of ongoing virus replication [20].

In this study, we aimed to gain further insights into OROV dynamics in human infection by using ag-RNA as a marker of actively replicating virus in different clinical samples, which were longitudinally collected from an OROV-infected Italian traveler returning from Cuba [15]. For this purpose, the roles of g- and ag-RNA as surrogate markers of ongoing OROV replication were preliminarily established via in vitro experiments, which were based on Vero E6 cells infected with a well-characterized viral isolate [21]. The forward and reverse primers used in these experiments were based on the primer pair designed by Naveca et al. [22], targeting the RNA S segment.

## 2. Materials and Methods

### 2.1. Clinical Samples

Whole blood, serum, urine, and semen samples from a patient with an OROV acute infection [15] were longitudinally collected for both diagnostic and research purposes after 4 (T1), 9 (T2), 15 (T3), 31 (T4), 57 (T5), 86 (T6), 135 (T7), and 177 (T8) DSO. All samples were processed on the same day of collection in order to minimize the risk of viral target degradation.

### 2.2. In Vitro OROV Infection

Vero E6 cells (CRL-1586™, American Type Culture Collection, ATCC^®^, Manassas, Virginia, USA) were maintained in Modified Eagle Medium (MEM, Life Technologies Europe, The Netherlands) supplemented with 10% heat-inactivated Fetal Calf Serum (FCS, Life Technologies Europe, Bleiswijk, Netherlands) at 37 °C in a humidified atmosphere with 5% CO_2_. To perform OROV infection, under BSL-3 conditions, the Vero E6 cells’ semiconfluent monolayer (80%) was inoculated with the recently identified Oropouche viral isolate, OROV_IRCCS-SCDC_1/2024_ [21] (GenBank Accession ID: S segment: PP952117; M segment: PP952118; and L segment: PP952119), at a multiplicity of infection (MOI) of 1. After viral adsorption at 37 °C for 1 h, the viral inoculum was removed, the cells were washed twice with phosphate-buffered saline, and then Minimum Essential Medium containing 2% Fetal Calf Serum was added. The total well contents (cells, supernatant, and total lysate), cell monolayers (cell-associated), and supernatants were harvested at different time points (6, 24, and 48 h post-infection, hpi) for the analysis of total RNA as well as g- and ag-RNA OROV S segments. For this aim, we used an approach that had been used in a previous study to investigate infection by Ebola virus, another negative-sense RNA virus [23]. Uninfected cells were used as a negative control.

### 2.3. Molecular Analyses

#### 2.3.1. RNA Extraction

RNA was extracted from the whole blood and urine using the EZ1^®^ Advanced XL System with the EZ1 DSP Virus Kit (Qiagen, Milan, Italy); both matrices were pre-diluted 1:4 (V:V) with PBS and then mixed with ATL buffer (Qiagen, Milan, Italy) following the manufacturer’s instructions. RNA from serum was isolated using the QIAamp Viral RNA Mini kit (Qiagen, Milan, Italy) following the manufacturer’s instructions. Semen was pre-diluted in 1:1 (V:V) with PBS, and RNA was then extracted using the InGenious^®^ magnetic beads-based system SP200 (ELITechGroup Molecular Diagnostics, Puteaux, France). An exogenous internal control (IC, AltoStar^®^ Internal Control 1.5, Altona Diagnostics, Hamburg, Germany) was added to the extraction step of all samples following the manufacturer’s instructions.

#### 2.3.2. One-Step Real-Time RT-PCR

All one-step real-time RT-PCRs were performed on the CFX96 Touch Real-Time PCR instrument (Biorad, Milano, Italy). Total OROV-specific RNA was amplified using previously described primers (OROV-FNF and OROV-FNR) and probe (OROV-FNP) [22], using the FlexStar^®^ RT-PCR Kit Amplification mix 1.5 (Altona Diagnostics, Hamburg, Germany, OROV cycle threshold (Ct) cut-off value < 38) and the LDT RT-PCR Detection Mix 1.5 (Internal Control amplification cut-off Ct value ≤ 34). Negative and positive controls were included in each real-time RT-PCR run; the Oropouche viral isolate, OROV_IRCCS-SCDC_1/2024_, was used as the positive control.

#### 2.3.3. cDNA Synthesis and Establishment of Real-Time PCR Specific for g-RNA and ag-RNA

The assay design to measure OROV-specific g-RNA and ag-RNA, including both the replication intermediate (cRNA) and messenger RNA (mRNA), was similar to the one previously used for another negative-sense RNA virus, i.e., Ebola virus [23]. Specifically, 2 μM of S segment OROV-FNR or OROV-FNF was used during the reverse transcription step with the SuperScript IV First-strand Synthesis System (Invitrogen, San Giuliano Milanese, Italy) following the manufacturer’s instructions. The synthesized cDNAs were treated with 1 μL of RNase H (2 U/μL) for 20′ at 37 °C to remove any remaining viral RNA. The genomic and antigenomic cDNAs were then amplified using the above-described OROV real-time RT-PCR, omitting the initial reverse transcription step.

#### 2.3.4. ddPCR

The ddPCR absolute quantification procedure for total, g-, and ag-RNA was performed on cDNAs obtained from both the in vitro infection experiments and clinical specimens, which were amplified with the same primers/probe set targeting the S segment of the OROV genome, as described above. Droplet generation and digital fluorescence reading were conducted with the Biorad QX200 Droplet Digital system. The amplification of both genomic and antigenomic cDNAs was performed with the ddPCR Supermix for Probes (No dUTP) Kit (Biorad, Milan, Italy) under the following cycling protocol: 95 °C for 10′ for enzyme activation, 95 °C for 30 s for denaturation, 60 °C for 60 s for annealing/extension for 50 cycles, 98 °C 10′ for enzyme deactivation, followed by 30′ at a 4 °C hold. The fluorescence collection data were analyzed using the QXManager 1.2 Standard Edition Software (Biorad, Milan, Italy) and expressed as copies/reaction values. The limit of detection was 1 copy/μL, as previously described [24]. For absolute quantification of total OROV RNA by ddPCR, cDNA from the in vitro infection experiments was synthesized and analyzed with the One-Step RT-ddPCR Advanced Kit for Probes (Biorad, Milan, Italy), as previously described [24].

### 2.4. Ethic Statement

This study was conducted in accordance with the ethical principles of the Declaration of Helsinki. Ethical clearance was obtained from the local Ethics Committee (Comitato Etico per la Sperimentazione Clinica dell’Area Sud Ovest Veneto), Prot. n. 68600 of 10/12/2024; the patient signed a written informed consent.

## 3. Results

### 3.1. OROV-RNA in Longitudinally Collected Clinical Samples

**Figure 1:**
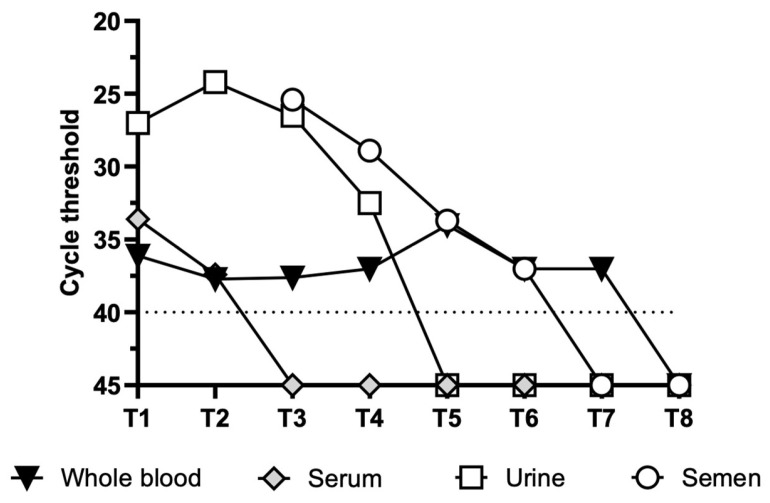
Time course of OROV total RNA levels in different clinical samples collected at different time points, starting from symptom onset. Viral RNA levels are expressed as cycle threshold (Ct) values of S segment sequence amplification. The dashed line represents the real-time RT-PCR limit of detection (Ct: 40).

Different clinical samples were longitudinally collected over a period of 4 to 177 days, starting from symptom onset (DSO), and tested for the presence of OROV total RNA using real-time RT-PCR (see Figure 1 and Table S1 in Supplementary Materials for cycle threshold values).

In the urine samples, OROV RNA was detected from 4 to 31 DSO (T1–T4) and remained negative at subsequent timepoints until T8 (177 DSO). On the other hand, whole blood maintained a low but constant viral load until T7 (135 DSO), whilst serum RNA was undetectable at T3 (15 DSO).

The semen samples tested positive (Ct 25.2) from the first analyzed sample (T3, 15 DSO) to 86 DSO (T6, Ct 37).

### 3.2. Time Course of g- and ag-RNA in Cells Infected with OROV In Vitro

To explore whether the presence of viral RNA in the samples collected longitudinally from different body sites was due to OROV replication, we employed the strategy of using the presence of ag-RNA (including both the replication intermediate and viral mRNA) as a marker of active viral replication. This approach was used in a previous study investigating infection caused by Ebola virus, another negative-sense RNA virus [23]. We previously validated the reliability of ag-RNA as a surrogate marker to support evidence of active viral replication using an in vitro model of infection based on OROV-infected Vero E6 cells.

Vero E6 cells were infected with the OROV_IRCCS-SCDC_1/2024_ isolate at multiplicity of infection (MOI) 1, and OROV g-RNA and ag-RNA were measured at different time points in the total, cell-associated, and released compartments using the newly established assays based on ddPCR (see Section 2).

**Figure 2:**
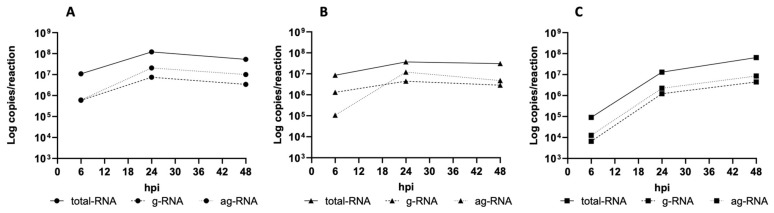
Time course of total, g-, and ag-RNA in OROV-infected Vero E6 cells established with the ddPCR method. (**A**) Lysate-associated total, g-, and ag-RNA; (**B**) cell-associated total, g-, and ag-RNA; (**C**) total, g-, and ag-RNA in the released compartment (supernatant). The results are expressed as copies/reaction.

As shown in Figure 2 (and Table S2 in Supplementary Materials for absolute ddPCR copies/reaction values), the time course of both parameters indicated a peak in cell-associated molecules at 24 hpi and a steady increase for molecules shed into the supernatant up to 48 hpi.

Notably, at both 24 and 48 hpi, the amounts of ag-RNA were slightly higher than those of g-RNA in both the cell-associated and released compartments. The observed patterns of g- and ag-RNA distributions offer confidence for the use of ag-RNA as an indirect marker of ongoing OROV replication.

### 3.3. Time Course of g- and ag-RNA in Longitudinally Collected Clinical Samples

**Figure 3:**
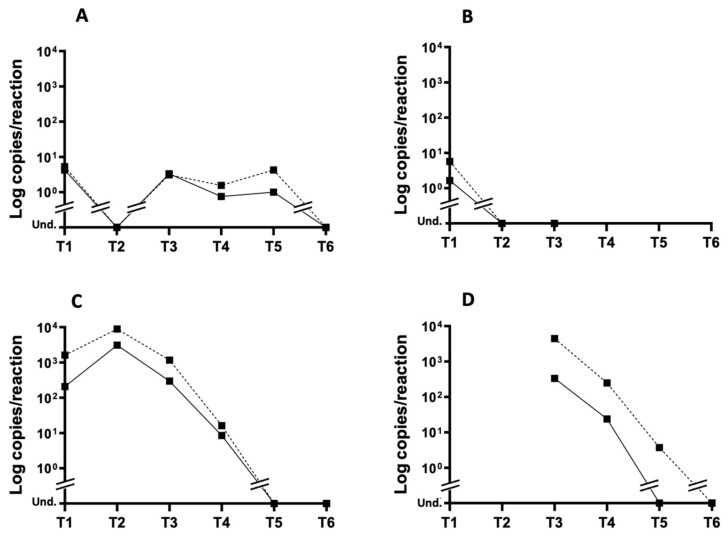
Time course of OROV g- and ag-RNA levels in longitudinally collected clinical samples. OROV g- (continuous line) and ag-RNA (dashed line) levels established by ddPCR: (**A**) g- and ag-RNA in whole blood; (**B**) g- and ag-RNA in serum; (**C**) g- and ag-RNA in urine; (**D**) g- and ag-RNA in semen. The results are expressed as copies/reaction. Und: undetectable. Double lines indicate a break in the continuity of the values on the axis.

Based on the results of the in vitro experiments, we explored the presence of g- and ag-RNA in samples that were longitudinally collected from an OROV-infected patient by applying the newly established ddPCR method. The results are illustrated in Figure 3. The absolute ddPCR copies/reaction values are reported in Supplementary Materials, Table S3.

The time course of g- and ag-RNA measured by ddPCR is consistent with the time course of total OROV RNA measured by real-time RT-PCR (Figure 1). Considering the separate measurements of g- and ag-RNA, the results indicate higher levels of ag-RNA than g-RNA, which was also observed in the in vitro experiments. Notably, in both urine and semen, OROV ag-RNA levels were higher than g-RNA levels at all time points, reaching undetectable levels at T5 and T6, respectively.

As shown in Supplementary Table S3, the largest difference in the amounts of g- and ag-RNA was observed at T1 in urine (more than seven times higher) and at T3 in semen (13 times higher). Unfortunately, no earlier samples were available for this matrix. Moreover, while it was possible to isolate the Oropouche virus from semen in culture at T3, it was not possible to obtain viral isolate from urine, as previously described [15].

The amounts of g- and ag-RNA detected in whole blood until T5 ranged between 1 and 10 copies/reaction, while in serum, both parameters were detectable in the same range of values only at T1. Retesting the whole blood, urine, serum, and semen samples with a real-time PCR protocol adapted to amplify the cDNA of g- and ag-RNA provided results that are substantially consistent with those measured by ddPCR (Supplementary Table S4).

## 4. Discussion

Since late 2023, more than 16,000 locally acquired cases of Oropouche virus infection have been reported in the Americas [25]. The first cases of travel-associated Oropouche fever were observed in the United States and Europe [7,8]. As with other arthropod-borne diseases, deforestation, climate change, and growing urbanization influence the distribution of OROV and increase the risk of its spread to new geographic regions [26]. Although this disease was previously known to be non-severe, two fatal cases of OROV infection have recently been reported in Brazil [27]. Evidence of its impact on pregnancy has recently emerged [10,28], with the cause attributed to vertical transmission. Moreover, organ transplants, transfusions, and semen donation may be influenced by previous arboviral infection events [29,30]. Recent findings have pointed to two further aspects that deserve attention: the detection of replication-competent virions in semen [16] and the longer-than-anticipated persistence of OROV RNA in different biological matrices [13,14]. These recent findings clearly highlight how little we know about the pathogenesis of this neglected arboviral infection in terms of transmission and shedding routes, persistence time, potential immunological sanctuaries, and impact at the pathogenic and clinical levels.

Hence, we explored the persistence of viral RNA in clinical samples that were longitudinally collected (from 4 to 177 DSO) from an immunocompetent adult who contracted OROV infection during a short visit to Cuba in July 2024. In particular, whole blood, serum, urine, and semen samples were collected and analyzed until all samples tested negative for total OROV RNA. We observed that viral RNA rapidly decayed in serum but persisted longer in other clinical samples; in particular, it was detectable (although at low levels) in whole blood for up to 135 DSO and in semen and urine at higher levels for up to 86 and 31 DSO, respectively.

To unravel whether persistent viral RNA detection in multiple clinical sample matrices was due to active viral replication or to the presence of vestigial traces of the viral genome, we established a quantitative assay that selectively measured viral g- and ag-RNA, designed based on the S segment of the OROV genome. To gain confidence in the reliability of this assay with ag-RNA as a surrogate marker of ongoing viral replication, we applied the newly established ddPCR in an in vitro time-course experiment using Vero E6 cells infected with OROV, and we assessed the kinetics of g- and ag-RNA in the culture compartments (supernatants and cell lysates).

The results of the in vitro infection experiment supported the use of ag-RNA as a surrogate marker of ongoing viral replication. In fact, as shown in Figure 2 and Table S2, the ag-RNA levels (including positive-sense viral RNA present in both the replication intermediate and viral mRNA) exceeded OROV-specific g-RNA levels, peaking at 24 hpi in the cellular compartment, as expected in the active replication phase. Subsequently, a slight decline was observed at 48 h. In the supernatant, the g- and ag-RNA levels steadily increased during the experiment as a consequence of viral shedding and cell lysis, respectively.

We subsequently explored the presence of g- and ag-RNA in the longitudinally collected clinical samples. The time course of OROV g- and ag-RNA levels from 4 to 177 DSO consistently showed higher amounts of ag-RNA than g-RNA. The presence of OROV ag-RNA in urine was higher than that of g-RNA at all time points, reaching undetectable levels at 31 DSO, with the largest difference in levels observed at 4 DSO (more than 7 times higher). In the semen samples, the ag-RNA levels remained higher than those of g-RNA, with the latter reaching levels near the detection limit earlier at 31 DSO, compared with 57 DSO for ag-RNA. The largest difference in the amounts of g- and ag-RNA was observed at 15 DSO (13 times higher). At this time point, the virus was also isolated in cell culture, as previously reported [15] (Figure 3 and Tables S3 and S4). To date, with the exception of a few cases of vertical transmission, no cases of inter-human transmission of OROV have been documented. However, the results of this study highlight potential transmission through the sexual route.

The main limitation of our study is that we analyzed clinical samples from only one patient, and the small sample size could affect the generalizability and representativeness of the results. Therefore, investigation with more clinical cases will be necessary to provide greater statistical power and robustness to our findings. Despite this limitation, our study opens a way for more in-depth investigation into OROV pathogenesis and transmission routes, especially sexually related pathways. It is worth noting that the Schmallenberg virus, another *Orthobunyavirus* genus member transmitted by biting midges to ruminants, has been isolated from bovine semen up to 3 months post-infection and may cause severe congenital infection of the fetus if acquired during pregnancy [31,32].

In whole blood, g- and ag-RNA levels between 1 and 10 copies/reaction were detected until 57 DSO, while in serum, both parameters were only detectable at 4 DSO.

The presence of OROV RNA in peripheral blood leukocytes during the acute phase and its persistence until 95 DSO in whole blood has previously been reported [14,33].

The long persistence in blood at a low viral load could indicate the presence of viral infection with low replicative activity in this body compartment.

As reported by Amorim and colleagues, OROV can generate a short-lasting productive infection in in vitro infected human peripheral blood mononuclear cells (PBMCs), as well as in T, B, and monocyte cell lines, although less efficiently. However, its ability to infect and replicate in human PBMCs from healthy individuals is restricted [20]. These findings suggest that OROV could use PBMCs as a Trojan horse for its dissemination into different tissues, and the infection may even reach the central nervous system [34].

Regarding the mechanisms that enable OROV persistence in cells, it is known that the orthobunyavirus NSs protein plays a protective role by acting as an interferon (IFN) antagonist and by binding to viral RNA to prevent its recognition by innate immune sensors such as RIG-I (retinoic acid-inducible gene-I) [35]. Through these two mechanisms, the NSs protein could help OROV evade the immune system and persist [36]. Studies examining the in vitro kinetics of Rift Valley Fever phlebovirus (RVFV) viral RNA accumulation indicated that the S segment ag-RNA is packaged into RVFV particles and transcribed to synthesize NSs mRNA immediately following infection, resulting in the expression of NSs at the early stages of infection [37], which could possibly lead to more efficient viral replication by interfering with RIG-I recognition [38].

Furthermore, negative-strand RNA viruses, such as measles, Ebola, and influenza A viruses, are able to establish persistent in vitro infection due to the presence of an excess of defective viral genomes that compete with the replication of non-defective genomes, reducing the extent of cellular damage and allowing the virus to persist in the host [39,40,41,42,43].

## 5. Conclusions

Our results, based on the measurement of ag-RNA, confirm and extend previous observations from our group and other researchers, suggesting that OROV may persist for a prolonged time in an actively replicating status in multiple body sites.

These findings may guide the choice of the most appropriate samples to collect for diagnostic purposes, considering that the recommended matrix (i.e., serum) seems to be suboptimal when compared with other matrices, such as whole blood or urine.

Further studies are needed to generalize our findings and to establish their pathogenetic implications, as well as the implications for transmission dynamics.

## Data Availability

All data are available in the text and Supplementary Materials section.

## Supplementary Materials

The following supporting information can be downloaded at https://www.mdpi.com/article/10.3390/v17060852/s1, Table S1: Cycle threshold of total OROV RNA in longitudinally collected clinical samples measured by real-time RT-PCR; Table S2: Time course and compartmentalization of total-RNA, g-RNA, and ag-RNA measured by ddPCR in OROV-infected Vero E6 cells; Table S3: OROV g- and ag-RNA measured by ddPCR in longitudinally collected clinical samples; Table S4: Cycle threshold of OROV g-RNA and ag-RNA in longitudinally collected clinical samples measured by real-time RT-PCR.

## Abbreviations

The following abbreviations are used in this manuscript:

OROV	Oropouche virus (OROV)
PAHO	Pan American Health Organization
WHO	World Health Organization
IRCCS_SCDC	IRCCS Sacro Cuore Don Calabria
ssRNA-	Negative-sense single-stranded RNA segments
DSO	Days from symptom onset
L	Large
M	Medium
S	Small
L Protein	RNA polymerase
G	Envelope glycoproteins
NS	Non-structural
N	Nucleocapsid
ag-RNA	Antigenomic RNA
g-RNA	Genomic RNA
RdRp	RNA-dependent RNA polymerase
cRNA	Replication intermediate RNA
mRNA	Messenger RNA
MOI	Multiplicity of infection
PBMC	Peripheral blood mononuclear cell
IFN	Interferon
RIG-I	Retinoic acid-inducible gene-I
RVFV	Rift Valley Fever phlebovirus
