# Supplementary material for: Persistence and Active Replication Status of Oropouche Virus in Different Body Sites: Longitudinal Analysis of a Traveler Infected with a Strain Spreading in Latin America
Source: Viruses. 2025 Jun 16;17(6):852. doi: 10.3390/v17060852 (PMC12197401; doi:10.3390/v17060852)
Supplement: Supplementary file 1 [file viruses-17-00852-s001.zip › viruses-3651951-supplementary.pdf]

Supplementary material

Supplementary tables

Table S1: Cycle threshold of total OROV RNA measured by *real time* RT-PCR in longitudinally collected clinical samples.

Time point (DSO)	T1 (4)	T2 (9)	T3 (15)	T4 (31)	T5 (57)	T6 (86)	T7 (135)	T8 (177)
				Cycle threshold				
**Whole blood**	36.1	37.7	37.6	37.0	34.0	37.0	37.0	>40
**Serum**	33.6	37.4	>40	>40	>40	>40	>40	>40
**Urine**	27.0	24,2	26.5	32.5	>40	>40	>40	>40
**Semen**	NA	NA	25.4	28.9	33.7	37	>40	>40

Ct values >40 are considered negative. NA: sample not available.

Table S2: Time course and compartmentalization of total-RNA, g-RNA and ag-RNA measured by ddPCR in OROV infected Vero E6 cells

		6 hpi	24 hpi	48 hpi
			copies/reaction	
**Total**	total-RNA	10,849,384	120,458,660	53,549,447
	g-RNA	592,928	7,462,079	3,398,699
	ag-RNA	612,140	20,880,150	10,104,752
**Cells**	total-RNA	8,607,697	36,802,171	30,887,626
	g-RNA	1,306,832	4,455,130	2,877,135
	ag-RNA	106,364	12,106,738	4,769,980
**Supernatant**	total-RNA	90,174	13,091,281	64,578,801
	g-RNA	6,603	1,228,860	4,454,850
	ag-RNA	12,703	2,237,209	8,558,205

Table S3: OROV g- and ag-RNA measured by ddPCR in longitudinally collected clinical samples.

Time point (DSO)		T1 (4)	T2 (9)	T3 (15)	T4 (31)	T5 (57)	T6 (86)
				Copies/reaction			
**Whole blood**	g-RNA	4.3	NT	3.3	1.0	1.0	NT
	ag-RNA	5.3	NT	3.1	1.6	4.3	NT
**Serum**	g-RNA	1.7	NT	NT	NT	NT	NT
	ag-RNA	5.7	NT	NT	NT	NT	NT

**Urine**	g-RNA	207.8	3079.5	296.0	8.5	NT	NT
	ag-RNA	1613.5	8838.8	1163.7	16.2	NT	NT
**Semen**	g-RNA	NA	NA	334.7	23.8	NT	NT
	ag-RNA	NA	NA	4474.8	247.5	3.7	NT

ND: not detected; NA: not available; NT: not tested.

Table S4: Cycle threshold of OROV g-RNA and ag-RNA measured by *real time* RT-PCR in longitudinally collected clinical samples.

**Time point (DSO)**		**T1 (4)**	**T2 (9)**	**T3 (15)**	**T4 (31)**	**T5 (57)**	**T6 (86)**	**T7 (135)**
				**Cycle Threshold**				
**Whole blood**	g-RNA	35.67	>40	35.06	37.2	36.2	>40	NT
	ag-RNA	34.43	39.4	34.68	35.9	35.3	>40	NT
**Serum**	g-RNA	37.34	39.62	>40	NT	NT	NT	NT
	ag-RNA	35.16	44.41	>40	NT	NT	NT	NT
**Urine**	g-RNA	28.11	25.29	29.23	33.4	>40	NT	NT
	ag-RNA	27.10	23.21	26.60	33.3	>40	NT	NT
**Semen**	g-RNA	NA	NA	27.42	31.2	>40	>40	NT
	ag-RNA	NA	NA	24.28	28.2	36.5	>40	NT

Ct values >40 are considered negative. NA: sample not available; NT: not tested.
